# COLORECTAL CANCER: ASSOCIATION BETWEEN SOCIODEMOGRAPHIC VARIABLES AND THE ADHERENCE TO CANCER SCREENING

**DOI:** 10.1590/0102-672020230002e1729

**Published:** 2023-05-12

**Authors:** Bruna Elisa Catin KUPPER, Fabio Oliveira FERREIRA, Wilson Toshihiko NAKAGAWA, Vinicius Fernando CALSAVARA, Thiago Celestino CHULAM, Ademar LOPES, Samuel AGUIAR-JUNIOR

**Affiliations:** 1 AC Camargo Cancer Center, Colorectal Cancer Department – São Paulo (SP), Brazil;; 2 Cedars-Sinai Medical Center, Samuel Oschin Comprehensive Cancer Institute – Los Angeles (CA), USA;; 3 AC Camargo Cancer Center, Department of Prevention and Early Diagnosis – São Paulo (SP), Brazil.

**Keywords:** Colorectal Neoplasms, Early Detection of Cancer, Mass Screening, Disease Prevention, Colonoscopy, Neoplasias Colorretais, Detecção Precoce de Câncer, Programas de Rastreamento, Prevenção de Doenças, Colonoscopia

## Abstract

**BACKGROUND:**

Colorectal cancer (CRC) is a worldwide health problem whose control depends on public policy establishment and effective prevention and screening programs. In Brazil, there are few studies related to adherence to screening methods.

**AIMS:**

The aim of this study was to evaluate the association between demographic and socioeconomic to adherence to CRC screening with fecal immunochemical test (FIT) among average-risk individuals for CRC.

**METHODS:**

In this prospective cross-sectional study, conducted between March 2015 and April 2016, 1,254 asymptomatic individuals aged 50–75 years, participating in a hospital screening campaign in Brazil, were invited to participate in the study.

**RESULTS:**

The adherence rate to FIT was 55.6% (697/1,254). In the multivariable logistic regression analysis, patients aged 60–75 years (odds ratio (OR)=1.30; 95% confidence interval (CI): 1.02–1.66; p=0.03), religious belief (OR=2.04; 95% CI: 1.34–3.11; p<0.01), previous fecal occult blood test (OR=2.07; 95% CI: 1.55–2.76; p<0.01), and full/part-time working status (OR=0.66; 95% CI: 0.49–0.89; p<0.01) were independently associated with adherence to CRC screening.

**CONCLUSION:**

The results of the present study highlight the importance of considering the labor aspects when implementing screening programs, suggesting that campaigns conducted in the workplace and repeated over the years may be more effective.

## RESUMO

**RACIONAL:**

O câncer colorretal (CCR) é um problema de saúde mundial cujo controle depende do estabelecimento de políticas públicas e programas de prevenção e rastreamento eficazes. No Brasil existem poucos estudos relacionados à adesão métodos de rastreamento.

**OBJETIVO:**

Avaliar a associação de características sócio-demográficas à realização de testes de sangue oculto nas fezes do tipo imunoquimicomecanizado (FIM) em população de médio risco para o desenvolvimento de câncer colorretal.

**MÉTODOS:**

Estudo observacional transversal, com coleta prospectiva de dados. Entre março de 2015 e abril de 2016, 1.254 indivíduos assintomáticos, com idade entre 50 e 75 anos, foram consecutivamente selecionados a partir de uma campanha hospitalar de rastreamento para neoplasias.

**RESULTADOS:**

As taxas de adesão ao teste FIM foi 55.6% (697/1254). Na análise de regressão logística múltipla os fatores independentes associados à adesão ao rastreamento do CCR foram: Idade entre 60–75 anos (oddsratio (OR)=1.30; intervalo de confiança de 95% (IC): 1.02–1.66; p=0.03), crença religiosa (OR=2.04; 95%IC: 1.34–3.11; p<0.01), realização prévia de exame de sangue oculto nas fezes (OR=2.07; 95%IC: 1.55–2.76; p<0.01) e vínculo empregatício em período integral ou parcial (OR=0.66; 95%IC: 0.49–0.89; p<0.01).

**CONCLUSÃO:**

Este estudo enfatiza a importância de considerar aspectos laborais ao implementar programas de rastreamento do câncer colorretal e sugere que campanhas de rastreamento implantadas no ambiente de trabalho e de maneira repetida ao longo dos anos podem ser mais efetivas.

## INTRODUCTION

In Brazil, colorectal cancer (CRC) is currently the third most commonly diagnosed cancer. A heterogeneous distribution of CRC has been observed in Brazil due to socioeconomic differences among the various regions of the country. The incidence of CRC cases is greater in regions characterized by higher levels of development and greater population density^16^. In contrast with developed countries like the United States and Canada, the incidence of CRC tumors in Brazil and South America has been increasing over the past couple of years^7^. In Brazil, 55–70% of CRC patients are diagnosed at an advanced clinical stage, and this contributes to a worse prognosis^31^. According to the World Health Organization (WHO), more than 70% of all deaths related to cancer occur in countries with low or middle level incomes, and these include countries with limited resources for the prevention, diagnosis, and treatment of cancer^34^.

Many factors related to the natural history disease of CRC favor population screening, like the classical carcinogenesis evolution from adenoma to cancer over a period of 10 years and the high incidence and high mortality of this disease^5,22,30,35^.

The Centers for Disease Control of the United States of America (USA) has estimated that if all individuals older than 50 years of age in a population undergo screening, it will be possible to achieve a 60% reduction in deaths due to CRC^22^. According to the WHO, at least 70% of a target population needs to be screened in a screening program^9^. Regular screening has also been shown to reduce mortality due to CRC by 15–33% and reduce the incidence of CRC by approximately 20% when a colonoscopy examination is used to detect polyps^9,11,33^. However, lower adherence to screenings has been observed in low-income populations who do not have health insurance^23^.

Despite colonoscopy is considered the most effective screening test for CRC, a recent study conducted in Brazil presented that the number of colonoscopies performed by the Unified Health System between 2010 and 2018 did not follow the population growth to attend the population and diagnosis colorectal tumors, emphasizing the importance of implementing screening programs with a fecal immunochemical test (FIT) in asymptomatic individuals to optimize the resource and disponibility of colonoscopy methods^4^.

Adherence to fecal occult blood test (FOBT) and colonoscopy screenings has been shown to vary according to ethnicity, sociodemographic characteristics, and personal health beliefs^18,19^. These observations highlight the importance of studying a target population in order to promote efficient and appropriate CRC screening campaigns according to population characteristics.

A national colorectal screening program using FOBT has been established in many countries. In contrast, there is no national population-based screening for CRC in Brazil, and local data regarding screening adherence is limited, as only a few studies have examined screening campaigns conducted that used FOBT^12,19^. Similarly, very few studies have evaluated factors related to CRC screening adherence in Latin American cultures^1^.

Given the importance of detecting CRC in its early stages and the limited data available regarding factors that influence adherence to CRC screenings in the Brazilian population, the aims of the study were to assess the rate of CRC screening among average-risk Brazilians aged 50–75 years and identify demographic, socioeconomic, and clinical factors that are associated with the adherence of this population to FIT.

## METHODS

This observational, cross-sectional study prospectively collected data from March 2015 to April 2016. Individuals from a public health campaign for cancer screening conducted by a private tertiary hospital were invited to participate. In this program, an annual screening was performed for CRC with FOBT.

Patients who met the inclusion criteria were aged 50–75 years, were asymptomatic for colorectal disease, and had previously been evaluated by a physician of the screening program who requested that FOBT be performed. Exclusion criteria were any kind of hereditary CRC syndrome, a personal history of gastrointestinal cancer, and residing outside the metropolitan region of Sao Paulo, Brazil. After receiving informed consent from participants, a structured questionnaire was used to collect demographic and clinical information from each participant. This study was approved by the Ethics Institutional Review Board of the A.C. Camargo Cancer Center (number: 2027/15).

A nurse was responsible for describing the importance of undergoing screenings for CRC as well as how the screening tests were performed. All of the participants received one fecal immunochemical test (FIT) kit (Eiken Chemical Co., Ltd., Japan) and written instructions regarding the correct method for collecting, storing, and delivering a sample to the clinical laboratory. Return of samples via a mailing service was not accepted. Participants were instructed to submit the samples themselves to the lab within 15 days of collecting a stool sample.

The primary outcome of this study was adherence to a CRC screening program. This outcome was fulfilled if the test was correctly performed within a maximum of 30 days after the participant was recruited. A group of trained nurses was responsible for collecting the examinations and checking if they were performed adequately.

Samples were analyzed with OC-Auto^®^ Micro 80 IFOB Site Inspection equipment. A colonoscopy was recommended if the test was positive. Data were analyzed in 2016.

### Statistical analysis

Categorical variables were described by descriptive statistics and presented as frequency. The chi-square test was used to evaluate possible associations between adherence to FIT and demographic characteristics presented as categorical variables.

Continuous variables were presented as mean±standard deviation (SD). An unpaired Student’s *t*-test was used to compare differences in mean values between the adherent and nonadherent groups.

Univariable analyses were used to evaluate direct and independent associations between measures and adherence to FIT, with odds ratios (OR) and 95% confidence intervals (CI) reported.

A multivariable logistic regression model was applied to assess the strength of associations between adherence to FIT and its predictors while controlling for confounders. Variables were included in the final model if they were deemed to be clinically important or if they were found to have a p-value of <0.20 in the univariable analyses. OR with 95%CI values were presented in association with primary outcome data^28^.

The Hosmer-Lemeshow goodness-of-fit test was used to evaluate the fit of the model^10^.

A nomogram was generated to graphically determine the probability of FIT adherence based on sociodemographic and clinical variables^3,24^.

Statistical analyses were performed using SPSS version 24.0 for Windows software (SPSS Inc., Chicago, IL, USA) and R version 2.3 (R Foundation for Statistical Computing, Vienna, Austria). For all tests, the significance level was fixed at 0.05, and a two-tailed test was used.

## RESULTS

A total of 1,254 individuals who had a medical indication to undergo CRC screening with FOBT consented to participate in this study. These participants completed a structured questionnaire to provide demographic and clinical information and submitted a sample for FIT. Adherence to FIT was 55.6% (697/1,254) for the cohort.

Of the participants, 65.3% (824/1,254) were women with a mean age of 59.8 years (range: 50–75 years), with 53.9% (676/1,254) aged 50–59 years and having an average of 8.7 years (range: 0–17 years) of education. Approximately half (52.1%, 653/1,254) of the participants had completed less than high school or had received no formal education at all. Over half (55.6%, 688/1,254) of the participants reported being married or living with a partner, while 55.7% (698/1,254) reported having full-time or part-time working positions. Characteristics according to the adherent and nonadherent groups are summarized in Table 1. The chi-square test results and comparison between means of the continuous variables for both groups are also presented in Table 1.

**Table 1:** Characteristics of the adherent and nonadherent groups in our cohort.

Characteristics	Adherent (n=697) n (%)	Nonadherent (n=557) n (%)	p-value
Gender			
Male	240 (34.4)	190 (34.1)	0.9
Female	457 (65.6)	367 (65.9)	
Age, years – mean (SD)	60.6 (6.7)	58.8 (6.7)	0.001
Age – categorical variable (years)			
50–59	343 (49.2)	333 (59.8)	<0.001
60–75	354 (50.8)	224 (40.2)	
Race/ethnic group			
White	377 (54.1)	311 (55.8)	0.26
Black	63 (9.0)	48 (8.6)	
Mixed	175 (25.1)	152 (27.3)	
Indian	21 (3.0)	15 (2.7)	
Asiatic	61 (8.8)	31 (5.6)	
Education, years – mean (SD)	8.4 (4.3)	9 (4.2)	0.01
Education – categorical variable			
No formal education	20 (2.9)	14 (2.5)	0.17
Less than high school	362 (52.0)	257 (46.2)	
High school graduate	194 (27.9)	183 (32.9)	
Some college or more	120 (17.2)	102 (18.3)	
Total	696	556	
Working status			
Full/part time	355 (50.9)	343 (61.6)	<0.001
Homemaker	140 (20.1)	106 (19.0)	
Unemployed/retired	202 (29.0)	108 (19.4)	
Marital status			
Single/divorced/widowed	280 (40.7)	227 (41.3)	0.81
Married/cohabiting	408 (59.3)	322 (58.6)	
Total	688	549	
Religious beliefs			
Yes	653 (94.1)	493 (88.7)	0.001
No	41 (5.9)	63 (11.33)	
Total	694	556	
Distance between residence and hospital, km – mean (SD)	19.3 (10.7)	20 (10.5)	0.19
Distance between residence and hospital – categorical variable			
<12.3 km	189 (27.1)	126 (22.7)	0.21
12.3–18.7 km	177 (25.4)	135 (24.3)	
18.7–25.75 km	166 (23.8)	147 (26.4)	
>25.75 km	165 (23.7)	148 (26.6)	
Total	697	556	
Income, dollar – mean (SD)	359.8 (357.8)	379.6 (446.7)	0.40
Income – categorical variable ($/day)			
Extremely poor (<$2.50)	19 (45.2)	23 (54.8)	0.17
Moderately poor ($2.50–$4)	38 (50.7)	37 (49.3)	
Vulnerable ($4–$10)	295 (59.7)	199 (40.3)	
Middle class and rich (>$10)	293 (57.2)	219 (42.8)	
Total	645	478	
Health insurance			
Yes	148 (25.0)	80 (22.5)	0.39
No	445 (75.0)	275 (77.5)	
Total	593	355	
Family history of CRC			
Yes	68 (9.9)	50 (9.0)	0.15
No	619 (90.1)	505 (91.0)	
Total	687	555	
Previous FOBT			
Yes	196 (28.2)	85 (15.44)	<0.001
No	499 (71.8)	467 (84.6)	
Total	695	552	
Self-referred previous knowledge of FOBT			
Yes	283 (40.6)	234 (42.1)	0.59
No	414 (59.4)	322 (58.0)	
Total	697	556	

SD: standard deviation; CRC: colorectal cancer; FOBT: fecal occult blood testing.

Over half (58.7%, 736/1,254) of the participants reported no previous knowledge of the existence or purpose of FOBT, and less than half (48.6%, 610/1,254) related no previous knowledge of the existence or purpose of a colonoscopy. Among all of the participants, only 281 (22.4%) and 204 (16.3%) participants confirmed that they had undergone FOBT or a colonoscopy prior to this study, respectively. Among the remaining participants who had never undergone FOBT, 94.1% (892/954) indicated that they had not received a “doctor’s recommendation” for the test, and 6.9% (66/954) reported that they did receive a “doctor’s recommendation,” yet they refused to undergo the examination. A history of refusing a colonoscopy was also reported by 31 (3.0%, 31/1,032) participants who never had the examination, while 97% (1,001/1,032) of them had never received a “‘doctor’s recommendation” for a colonoscopy. Among the participants who previously underwent a colonoscopy, 48.0% (98/204) reported that the main reasons for undergoing the examination were abdominal pain, bleeding, or unusual bowel patterns; 37.2% (76/204) indicated that the examination was part of a check-up; and 14.7% (30/204) did not know why they had undergone the examination. Concerning a familial history of CRC, 118 (9.4%) participants reported a history of cancer. Among all of these described conditions, only a history of undergoing FOBT was found to be related to a higher adherence to CRC screening (Table 1).

p-values are associated with the t-test or χ^2^ test. Continuous variables were evaluated by independent t-test (italics), and χ^2^ test was used to investigate the association between categorical variable and adherence to FIT. Statistically significant differences are represented when p<0.05.

Independent associations between variables evaluated in the questionnaire and individuals’ adherence to FIT were determined by using a univariable logistic regression model (Table 2). Associations were observed between FIT and patients aged 60–75 years (OR=1.53; 95%CI: 1.22–1.92), history of previous FOBT (OR=2.16; 95%CI: 1.62–2.87), full/part-time working status (OR=0.55; 95%CI: 0.42–0.73), position as a homemaker (OR=0.70; 95%CI: 0.51–1.00), and religious belief (OR=2.03; 95%CI: 1.35–3.07).

**Table 2:** Univariable logistic regression models for the adherence to fecal immunochemical tests.

Variables	OR	95%CI		p-value
		Lower	Upper	
Age, years				
60–75	1.53	1.22	1.92	<0.001
50–59	Ref			
Gender				
Male	0.98	0.77	1.25	0.90
Female	Ref			
Self-referred previous knowledge of FOBT				
Yes	0.94	0.75	1.18	0.60
No	Ref			
Previous FOBT				
Yes	2.16	1.62	2.87	<0.001
No	Ref			
Distance between residence and hospital (km)				
<12.3	Ref			0.21
12.3–18.7	0.87	0.63	1.20	0.41
18.7–25.75	0.75	0.55	1.03	0.08
>25.75	0.74	0.54	1.02	0.07
Education				
No formal education	Ref			0.17
Less than high school	0.98	0.49	1.99	0.97
High school graduate	0.74	0.36	1.51	0.41
Some college or more	0.82	0.40	1.71	0.60
Family history of CRC				
Yes	1.11	0.76	1.63	0.59
No	Ref			
Working status				
Unemployed/retired	Ref			<0.001
Full/part time	0.55	0.42	0.73	<0.001
Homemaker	0.70	0.51	1.00	0.05
Income ($/day)				
Extremely poor (<$2.50)	Ref			0.17
Moderately poor ($2.50–$4)	1.24	0.58	2.65	0.57
Vulnerable ($4–$10)	1.79	0.95	3.38	0.07
Middle class and rich (>$10)	1.62	0.86	3.04	0.13
Marital status				
Married or cohabitating	0.97	0.77	1.22	0.82
Single or living alone	Ref			
Religious beliefs				
Yes	2.03	1.35	3.07	<0.001
No	Ref			
Health insurance				
Private health insurance	1.14	0.84	1.56	0.40
No health insurance	Ref			

OR: odds ratio; CI: confidence interval; FOBT: fecal occult blood testing; CRC: colorectal cancer. OR was computed based on the univariable logistic regression model. Data significant at p<0.05.

A multivariable logistic regression model was subsequently applied to evaluate variables with clinical importance and those identified in the univariate analysis (e.g., gender, age, education, previous FOBT, religion, working status, marital status, distance between the hospital, and income). The final model was identified by using the stepwise method (backward). The variables that were found to be independently associated with CRC screening included patient age (60–75 years) (OR=1.30; 95%CI: 1.02–1.66), religious belief (OR=2.04; 95%CI: 1.34–3.11), previous FOBT (OR=2.07; 95%CI: 1.55–2.76), and full/part-time working status (OR=0.66; 95%CI: 0.49–0.89) (Table 3). The model has a reasonable fit (Hosmer-Lemeshow of p=0.085).

**Table 3:** Multivariable logistic regression model for colorectal cancer screening with fecal immunochemical testing.

Variables	OR	Lower	Upper	p-value
Age, years				
60–75	1.30	1.02	1.66	0.03
50–59	Ref			
Religious beliefs				
Yes	2.04	1.34	3.11	0.001
No	Ref			
Previous FOBT				
Yes	2.07	1.55	2.76	<0.001
No	Ref			
Employment status				
Unemployed/retired	Ref			0.08
Full/part time	0.66	0.49	0.89	0.006
Homemaker	0.74	0.52	1.06	0.1

OR: odds ratio; FOBT: fecal occult blood testing. OR was computed based on the multivariable logistic regression model adjusted for gender, age, education, previous FOBT, religion, working status, marital status, distance between the hospital, and income.

**Figure 1:**
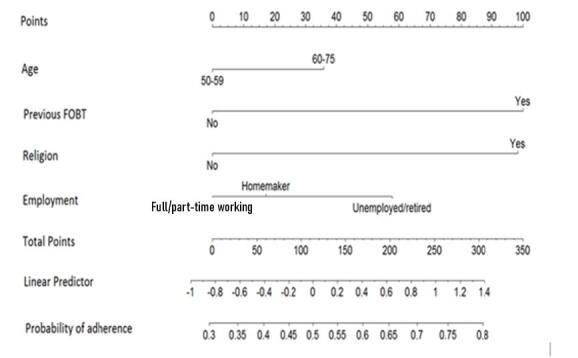
Nomogram estimating probability of adherence to fecal immunochemical tests. This nomogram was constructed to illustrate the probability of adherence to fecal immunochemical tests according to clinical, social, and economic characteristics evaluated in this study and analyzed using the multivariable logistic regression model.

A nomogram was constructed to illustrate the probability of adherence to FIT according to clinical, social, and economic characteristics evaluated in this study (Figure 1). A cumulative point score for all of the variables was matched with a corresponding number of points assigned to the individual scores of the variables, and then the cumulative point score for all of the variables was matched to a scale of adherence to FIT.

## DISCUSSION

In the average-risk group of individuals who were enrolled and evaluated in this tertiary hospital screening campaign in Brazil, young age, an absence of religious beliefs, no previous history of FOBT, and full/part-time working status were associated with an increased risk of not adhering to CRC screening with FIT. Accordingly, a study conducted with data obtained from the French national screening program and from many randomized trials conducted in the United States and the United Kingdom also found greater adherence to screening with FOBT among older individuals^9,11,26,29,32^.

We hypothesized that greater adherence is observed when individuals are exposed to CRC screenings over multiple years. This hypothesis is consistent with the identification of previous screening with FOBT as an independent variable in the multivariable analysis.

Multivariable analysis was performed in the present study and the nearly doubled probability of adherence observed for the group was previously screened with FOBT. We further hypothesized that greater adherence to CRC screening by older individuals is due to their knowledge of higher risk of being affected by CRC.

When a population that underwent multiple FOBT screenings was evaluated in subsequent years, the adherence of this population was found to increase by 8–17% after the second round of screening^2,27^. However, other studies that have evaluated the effect of repeat occult blood tests in national screening programs have not shown an increase in the percentage of adherence to screening in subsequent years^14^. It is possible that these inconsistent results are due to differences in the methodologies employed in these studies.

Work status was a variable that we considered in the present study. We observed that greater adherence to screening with FOBT was exhibited by individuals who were categorized as retired or unemployed. We hypothesized that this finding reflects a population of individuals who have a greater appreciation and awareness of cost-free health services, such as those offered by the screening campaign evaluated in the present study. In contrast, Greiner et al.^8^ did not identify the type of work activity as a factor associated with adherence to CRC screening in their study. This difference in the selection of variables examined may affect how adherence is evaluated. For example, in the Greiner study, adherence outcome was determined based on mail service, while in the present study, adherence was achieved when a sample prepared with a FIT kit was received and analyzed at our clinical laboratory within 30 days of participant recruitment^8^. It is also possible that the requirement in the present study that stool samples be collected during business hours contributed to the lower adherence rate of part-time or full-time workers enrolled in this study compared with retirees or those who were unemployed, since the latter two groups of individuals would be expected to have more time available. It is also important to note that this same sample collection strategy has been adopted by most services in Brazil.

In an observational, cross-sectional study of 1,352 adults older than 50 years of age that was conducted in Palestine by Qumseya et al.,^21^ attitudes and barriers related to low adherence to screening for CRC were also assessed^25^. This study found that a lack of knowledge regarding screening for CRC was independently associated with decreased adherence to screening. In addition, older age and being employed were variables associated with a decrease in adherence to FOBT screening^21^. Similarly, studies conducted in the United States have shown that individuals with limited knowledge about CRC have negative attitudes toward screening and are less likely to undergo FOBT, especially groups with lower health literacy^6,15^.

In the present study, participants were asked about their prior knowledge of CRC screening methods, although standardized instruments were not used to evaluate the knowledge presented. Therefore, a limitation of the present study is that the knowledge referred to may not be consistent with the actual knowledge presented. However, a history of undergoing screening tests may indicate a greater understanding/appreciation of CRC and its risks, which would support our hypothesis that higher adherence is associated with individuals who have previously undergone CRC screenings.

Income was not identified as a factor associated with adherence to screening in the present study. In addition, there was no statistically significant difference in the mean income of the groups that adhered or did not adhere to the screening opportunity. However, the majority (54.4%) of the participating individuals were categorized as extremely poor, moderately poor, or vulnerable according to the classification of the World Bank^13^, indicating that the participants of the present study mostly represented a low economic level population.

The nomogram used to summarize and illustrate our data for the population studied remains to be validated in future studies in order to confirm the present results and determine the applicability to other populations.

According to the WHO, the implementation of a national screening program for CRC with FOBT should be evaluated with regard to implementation costs, logistics, and its impact on the number of colonoscopies performed for positive screening results. Overall, the effectiveness of screening is related to high adherence rates. Thus, it is important and necessary for educational actions and adequate strategies to be developed that stimulate adherence to screening examinations^17^.

Perin et al. conducted a study in Brazil to evaluate the knowledge, attitudes, and practices of physicians, nurses, and health coordinators with roles related to CRC screening^20^. The authors found that only half of the physicians investigated ordered screening tests for asymptomatic and eligible patients, and only 30% of the physicians started screening patients aged 50–55 years. Thus, adherence to international and national recommendations for CRC screening is poor in Brazil.

These data highlight the importance of conducting future studies to validate the results summarized in the nomogram generated in this study, as well as the cost-effectiveness of CRC screening in Brazil. It is of great relevance to Brazilian society not only due to the high incidence of CRC in the population, but also because there have been few national studies of this nature conducted in Brazil. Studies of the risk perception, health literacy, and decision-making processes of target populations for screening in Brazil are also extremely important for the goal of advancing a structured national policy for CRC screening in Brazil that will be both adequate and effective.

## CONCLUSIONS

Based on the population examined, younger individuals with no religious beliefs who never underwent FOBT and had full/part-time working status exhibited an increased risk of not adhering to CRC screening with FIT. The present study highlights the importance of considering the labor aspects when implementing screening programs, suggesting that campaigns conducted in the workplace and repeated over the years may be more effective. Thus, these findings can help direct future early detection campaigns for CRC, focusing on obtaining better adherence of the population, adhering to screening with FIT. It may also facilitate the development of a national public policy program for CRC screenings.
